# Supplementary material for: Temporal Trends in HIV-1 Mutations Used for the Surveillance of Transmitted Drug Resistance
Source: Viruses. 2021 May 11;13(5):879. doi: 10.3390/v13050879 (PMC8150354; doi:10.3390/v13050879)
Supplement: Supplementary file 1 [file viruses-13-00879-s001.zip › viruses-1195520-supplementary.pdf]

Table S1. List of 113 PI-associated mutations included in one or more of four expert 2020 lists: prevalence in PI-naïve and PI-experienced persons before and after 2009.

Pos[1]	AA[1]	Lists		<2009											≥2009										
		2009	2020	%PI	%PI-Naive										%PI	%PI-Naive									
				(22,266)	ALL (61,067)	A (4,316)	B (33,132)	C (8,935)	01 (5,265)	02 (3,743)	D (1,497)	F (726)	G (2,036)	Other (1,438)	(10,534)	ALL (54,813)	A (3,209)	B (21,435)	C (10,194)	01 (10,189)	02 (3,616)	D (891)	F (794)	G (1,425)	Other (3,039)
10	C	1	1	0.0	0.0	0.0	0.0	0.0	0.0	0.0	0.0	0.0	0.0	0.0	0.0	0.0	0.0	0.0	0.0	0.0	0.0	0.0	0.0	0.0	0.0
10	F	4	4	8.6	0.2	0.1	0.3	0.1	0.1	0.0	0.3	0.0	0.1	0.5	6.7	0.2	0.2	0.3	0.1	0.2	0.1	0.5	0.0	0.0	0.0
10	I	4	3	31	8.5	14	8.9	2.3	10	6.0	6.1	9.1	11	26	16	8.4	20	9.2	2.2	7.7	7.7	6.6	10	19	15
10	M	1	1	0.2	0.3	0.6	0.0	0.8	0.3	0.1	0.2	0.1	1.5	1.4	0.3	0.5	0.8	0.2	1.2	0.2	0.5	0.0	0.3	2.5	2.0
10	R	3	1	0.4	0.0	0.0	0.0	0.0	0.0	0.0	0.0	0.0	0.0	0.0	0.2	0.0	0.0	0.0	0.0	0.0	0.0	0.0	0.0	0.0	0.0
10	V	4	3	11	4.8	8.5	3.4	1.8	5.9	11	8.1	33	0.5	7.6	11	5.9	12	4.4	1.7	5.9	12	8.6	50	1.6	4.8
11	I	4	4	3.0	0.6	0.5	0.3	0.5	0.5	3.3	0.5	0.0	1.1	1.4	1.4	0.8	1.1	0.3	0.5	0.6	4.6	0.2	0.6	1.6	0.3
11	L	1	1	0.7	0.0	0.0	0.0	0.1	0.0	0.0	0.1	0.0	0.0	0.0	0.2	0.0	0.2	0.0	0.0	0.0	0.0	0.1	0.0	0.1	0.0
15	A	1	1	0.0	0.0	0.0	0.0	0.0	0.0	0.0	0.0	0.0	0.0	0.0	0.0	0.0	0.0	0.0	0.0	0.0	0.0	0.0	0.0	0.0	0.1
15	V	1	1	33	28	15	22	85	12	10	17	75	17	32	41	33	19	25	81	14	16	18	60	22	25
16	E	3	1	6.2	9.4	20	4.2	7.2	25	23	9.0	26	4.5	16	13	14	24	6.2	9.1	26	31	9.8	30	6.0	19
20	I	4	2	8.4	9.9	2.7	0.1	0.1	2.7	95	0.7	0.4	98	8.9	6.2	11	5.4	0.2	0.1	1.9	95	2.4	3.1	97	9.8
20	M	4	3	3.3	0.5	0.1	0.4	0.6	0.1	0.6	1.3	1.3	0.9	1.8	1.7	0.5	0.1	0.6	0.6	0.0	0.5	2.7	2.3	0.6	1.1
20	R	4	3	17	8.2	17	2.8	19	16	1.7	12	43	0.0	18	18	13	20	3.0	22	27	2.3	13	31	0.4	11
20	T	4	3	5.3	0.1	0.1	0.1	0.1	0.1	0.1	0.2	0.0	0.0	0.8	4.1	0.1	0.0	0.1	0.1	0.2	0.1	0.1	0.0	0.0	0.0
20	V	3	2	1.1	0.1	0.0	0.0	0.0	0.0	1.5	0.2	0.0	0.6	0.2	0.5	0.1	0.1	0.0	0.0	0.0	1.4	0.0	0.0	0.9	0.5
**23**	**I**	2	2	1.3	0.0	0.2	0.0	0.0	0.0	0.0	0.1	0.0	0.0	0.0	1.1	0.0	0.0	0.0	0.0	0.0	0.0	0.0	0.0	0.0	0.0
24	F	2	1	0.6	0.0	0.1	0.0	0.1	0.0	0.0	0.1	0.0	0.0	0.0	0.3	0.0	0.0	0.0	0.0	0.0	0.0	0.1	0.0	0.0	0.0
**24**	**I**	4	4	5.6	0.0	0.0	0.0	0.0	0.0	0.0	0.1	0.0	0.0	0.0	2.5	0.0	0.0	0.0	0.0	0.0	0.0	0.0	0.0	0.0	0.0
24	M	0	1	0.1	0.0	0.0	0.0	0.0	0.0	0.0	0.0	0.0	0.0	0.0	0.1	0.0	0.0	0.0	0.0	0.0	0.0	0.0	0.0	0.0	0.0
**30**	**N**	4	4	6.2	0.0	0.0	0.0	0.0	0.0	0.0	0.0	0.0	0.0	0.0	2.4	0.0	0.0	0.0	0.0	0.0	0.0	0.0	0.0	0.0	0.0
**32**	**I**	4	4	5.0	0.0	0.0	0.0	0.0	0.0	0.0	0.0	0.0	0.0	0.0	2.5	0.0	0.0	0.0	0.0	0.0	0.0	0.0	0.0	0.1	0.0
32	ins	1	1	0.0	0.0	0.0	0.0	0.0	0.0	0.0	0.0	0.0	0.0	0.0	0.0	0.0	0.0	0.0	0.0	0.0	0.0	0.0	0.0	0.0	0.0
33	F	4	4	12	0.4	1.0	0.2	0.1	1.4	0.4	0.7	0.0	0.4	0.5	6.9	0.5	1.6	0.1	0.2	1.2	0.6	0.4	0.3	0.2	0.7
33	I	4	3	2.2	0.6	0.3	1.0	0.0	0.2	0.2	1.4	0.1	0.2	0.6	1.7	0.7	0.8	1.4	0.0	0.1	0.2	0.9	0.6	0.2	0.6
33	V	4	2	1.8	1.7	0.5	2.6	0.1	0.5	0.2	7.0	0.6	0.3	0.8	1.4	1.3	0.7	2.6	0.3	0.3	0.4	6.4	1.0	0.1	0.8
34	Q	1	1	2.6	0.1	0.0	0.0	0.0	0.0	0.5	0.0	0.0	0.7	0.0	1.3	0.1	0.0	0.0	0.0	0.0	0.7	0.0	0.0	0.4	0.3
35	G	3	1	1.1	0.2	0.1	0.1	0.1	0.1	1.5	0.1	0.1	0.3	1.2	0.7	0.2	0.1	0.1	0.1	0.1	1.4	0.2	0.0	0.4	0.7
35	N	1	1	1.6	0.6	1.0	0.1	0.2	1.7	2.6	0.3	0.7	2.9	0.3	1.9	1.0	3.0	0.1	0.3	1.1	3.8	1.9	0.9	3.1	1.5
36	I	4	2	49	51	98	17	84	97	97	63	97	98	79	62	62	97	18	85	97	97	71	96	98	69

36	L	3	2	2.6	1.3	0.4	1.0	3.6	0.3	0.9	2.5	0.7	0.3	1.8	2.5	1.4	0.6	1.2	3.6	0.2	0.8	1.3	0.8	0.5	1.0
36	V	3	2	2.1	1.0	0.4	0.4	4.7	0.6	0.2	0.5	0.1	0.2	1.2	1.4	1.1	0.2	0.4	3.4	0.4	0.3	0.6	0.8	0.1	2.7
38	W	1	1	0.2	0.0	0.0	0.0	0.0	0.0	0.0	0.0	0.1	0.0	0.0	0.1	0.0	0.0	0.0	0.0	0.0	0.0	0.0	0.0	0.0	0.0
41	T	1	1	0.1	0.0	0.0	0.0	0.1	0.0	0.1	0.1	0.0	0.1	0.3	0.1	0.0	0.0	0.0	0.1	0.0	0.2	0.0	0.0	0.0	0.1
43	R	1	1	2.4	2.7	1.4	3.0	1.3	2.4	5.6	1.6	3.7	3.1	5.0	3.0	3.2	2.7	3.4	1.3	2.7	7.8	4.5	2.3	4.1	3.9
43	T	3	3	5.6	0.1	0.1	0.1	0.1	0.0	0.2	0.1	0.1	0.1	1.2	3.0	0.1	0.1	0.1	0.1	0.0	0.2	0.0	0.0	0.0	0.4
**46**	**I**	4	4	21	0.3	0.3	0.3	0.2	0.3	0.2	0.1	0.0	0.0	0.2	13	0.3	0.3	0.4	0.1	0.6	0.2	0.1	0.4	0.2	0.0
**46**	**L**	4	4	9.2	0.3	0.3	0.4	0.1	0.3	0.1	0.2	0.1	0.4	0.3	3.4	0.4	0.2	0.3	0.1	0.9	0.1	0.0	0.4	0.0	0.6
46	V	1	1	0.5	0.0	0.0	0.0	0.1	0.1	0.1	0.0	0.0	0.0	0.0	0.4	0.1	0.0	0.1	0.0	0.1	0.1	0.0	0.1	0.0	0.1
**47**	**A**	4	4	0.4	0.0	0.0	0.0	0.0	0.0	0.0	0.0	0.0	0.0	0.0	1.0	0.0	0.0	0.0	0.0	0.0	0.0	0.0	0.0	0.0	0.0
**47**	**V**	4	4	4.5	0.0	0.0	0.0	0.0	0.0	0.1	0.0	0.0	0.1	0.0	2.0	0.0	0.0	0.0	0.0	0.0	0.0	0.0	0.0	0.1	0.0
48	A	2	2	0.5	0.0	0.0	0.0	0.0	0.0	0.0	0.0	0.0	0.0	0.0	0.2	0.0	0.0	0.0	0.0	0.0	0.0	0.0	0.0	0.0	0.1
48	L	0	1	0.1	0.0	0.0	0.0	0.0	0.0	0.0	0.0	0.0	0.0	0.0	0.0	0.0	0.0	0.0	0.0	0.0	0.0	0.0	0.0	0.0	0.0
**48**	**M**	2	2	0.5	0.0	0.0	0.0	0.0	0.0	0.0	0.0	0.0	0.0	0.0	0.2	0.0	0.0	0.0	0.0	0.0	0.0	0.0	0.0	0.0	0.0
48	Q	0	1	0.1	0.0	0.0	0.0	0.0	0.0	0.0	0.0	0.0	0.0	0.0	0.1	0.0	0.0	0.0	0.0	0.0	0.0	0.0	0.0	0.0	0.0
48	S	1	1	0.1	0.0	0.0	0.0	0.0	0.0	0.0	0.0	0.0	0.0	0.0	0.1	0.0	0.0	0.0	0.0	0.0	0.0	0.0	0.0	0.0	0.0
48	T	1	1	0.0	0.0	0.0	0.0	0.0	0.0	0.0	0.0	0.0	0.0	0.0	0.0	0.0	0.0	0.0	0.0	0.0	0.0	0.0	0.0	0.0	0.0
**48**	**V**	4	4	3.7	0.0	0.0	0.0	0.0	0.0	0.0	0.0	0.0	0.0	0.0	0.7	0.0	0.0	0.0	0.0	0.0	0.0	0.0	0.0	0.0	0.0
**50**	**L**	4	4	1.5	0.0	0.0	0.0	0.0	0.0	0.0	0.0	0.0	0.0	0.0	2.5	0.0	0.0	0.0	0.0	0.0	0.0	0.0	0.0	0.0	0.0
**50**	**V**	4	4	1.8	0.0	0.0	0.0	0.0	0.0	0.0	0.0	0.0	0.0	0.0	1.2	0.0	0.0	0.0	0.0	0.0	0.0	0.0	0.0	0.0	0.0
**53**	**L**	4	4	5.9	0.0	0.0	0.0	0.0	0.0	0.0	0.1	0.1	0.0	0.0	3.0	0.0	0.0	0.0	0.0	0.0	0.0	0.1	0.0	0.0	0.0
53	W	1	1	0.1	0.0	0.0	0.0	0.0	0.0	0.0	0.0	0.0	0.0	0.0	0.0	0.0	0.0	0.0	0.0	0.0	0.0	0.0	0.0	0.0	0.0
**53**	**Y**	3	2	0.4	0.0	0.0	0.0	0.0	0.0	0.1	0.0	0.0	0.0	0.0	0.2	0.0	0.0	0.0	0.0	0.0	0.2	0.1	0.0	0.0	0.0
**54**	**A**	4	4	1.3	0.0	0.0	0.0	0.0	0.0	0.0	0.0	0.0	0.0	0.0	0.4	0.0	0.0	0.0	0.0	0.0	0.0	0.0	0.0	0.0	0.0
**54**	**L**	4	4	3.0	0.0	0.0	0.0	0.0	0.0	0.0	0.0	0.0	0.0	0.0	1.4	0.0	0.1	0.0	0.0	0.0	0.0	0.0	0.0	0.0	0.0
**54**	**M**	4	4	2.3	0.0	0.0	0.0	0.0	0.0	0.0	0.0	0.1	0.0	0.0	0.6	0.0	0.0	0.0	0.0	0.0	0.0	0.0	0.0	0.0	0.1
**54**	**S**	4	3	0.7	0.0	0.0	0.0	0.0	0.0	0.0	0.0	0.0	0.0	0.0	0.1	0.0	0.0	0.0	0.0	0.0	0.0	0.0	0.0	0.0	0.0
**54**	**T**	4	4	0.8	0.0	0.1	0.0	0.0	0.0	0.0	0.0	0.0	0.0	0.2	0.2	0.0	0.0	0.0	0.0	0.0	0.0	0.0	0.1	0.0	0.0
**54**	**V**	4	4	23	0.0	0.0	0.0	0.0	0.0	0.0	0.0	0.0	0.0	0.0	12	0.0	0.0	0.0	0.0	0.0	0.0	0.0	0.0	0.0	0.0
58	E	4	3	6.3	0.3	0.1	0.4	0.4	0.0	0.0	1.2	0.1	0.1	0.2	4.3	0.4	0.1	0.6	0.5	0.0	0.0	0.7	0.5	0.3	0.4
60	E	2	2	12	9.0	5.2	8.9	17	2.8	0.6	16	11	2.1	37	13	15	6.2	18	19	2.9	1.0	15	27	2.4	32
62	V	3	3	34	17	6.3	27	4.6	4.7	1.4	19	19	3.2	20	25	17	7.9	33	5.1	5.5	2.2	21	23	6.3	16
63	P	3	2	63	40	15	55	36	12	19	35	9.1	20	14	49	37	17	54	37	17	22	39	9.6	24	33
64	L	1	1	2.9	3.6	1.2	4.5	1.9	0.6	7.3	3.1	2.6	3.6	2.4	3.4	3.6	2.3	4.7	2.2	0.6	9.8	5.2	2.8	5.1	2.9
64	M	2	2	1.7	2.6	2.0	1.6	1.6	0.8	13	0.8	2.1	9.6	3.6	2.2	3.3	3.4	1.9	1.9	0.6	18	0.9	1.9	13	2.1
64	V	2	3	18	11	0.8	16	0.5	0.4	1.7	66	0.6	0.4	13	13	7.1	1.2	14	0.8	0.6	1.8	57	1.5	0.7	5.5
66	V	0	1	1.1	0.1	0.1	0.1	0.1	0.0	0.4	0.1	0.0	0.1	1.4	0.4	0.1	0.1	0.0	0.1	0.0	0.2	0.0	0.1	0.4	0.4

69	I	1	1	0.1	0.0	0.0	0.0	0.0	0.1	0.1	0.1	0.0	0.0	0.0	0.1	0.0	0.1	0.0	0.0	0.1	0.1	0.0	0.0	0.1	0.0
69	K	3	2	15	41	96	1.5	98	97	96	2.8	1.4	94	61	41	54	92	2.6	97	96	96	6.4	3.7	89	58
69	N	1	1	1.0	0.5	0.1	0.7	0.1	0.1	0.2	0.3	0.1	0.3	0.2	0.2	0.3	0.3	0.6	0.0	0.2	0.3	0.1	0.6	0.4	0.4
69	Q	1	1	3.3	2.4	2.1	2.9	1.1	0.7	1.0	5.1	8.4	1.4	11	4.2	2.8	4.4	4.0	1.3	0.7	1.1	16	7.3	1.1	6.7
69	R	1	2	1.5	0.6	0.7	0.4	0.3	1.0	1.2	0.3	1.0	4.0	1.2	1.0	0.9	1.3	0.4	0.4	0.9	2.3	0.3	0.6	8.4	0.8
69	Y	1	1	2.2	1.9	0.0	2.5	0.0	0.0	0.0	18	7.2	0.0	1.2	1.9	1.4	0.2	2.3	0.0	0.0	0.0	9.7	17.9	0.0	0.7
71	I	4	2	3.2	0.1	0.0	0.1	0.0	0.0	0.0	0.0	0.0	0.0	0.0	1.7	0.1	0.0	0.3	0.0	0.0	0.0	0.0	0.0	0.0	0.0
71	L	3	2	0.4	0.0	0.0	0.0	0.0	0.0	0.0	0.0	0.0	0.0	0.0	0.2	0.0	0.0	0.0	0.0	0.0	0.0	0.0	0.0	0.0	0.0
71	T	4	3	9.9	4.7	0.1	8.2	1.0	0.2	0.2	1.9	0.1	0.4	0.3	6.3	4.7	0.2	11	1.0	0.3	0.3	3.0	0.5	0.5	2.5
71	V	4	2	27	3.3	0.0	5.9	0.2	0.3	0.2	0.3	0.1	0.3	0.5	12	3.5	0.2	8.0	0.3	0.2	0.1	0.6	0.1	1.1	6.1
**73**	**A**	4	2	0.6	0.0	0.0	0.0	0.0	0.0	0.0	0.0	0.0	0.0	0.0	0.2	0.0	0.0	0.0	0.0	0.0	0.0	0.0	0.0	0.0	0.0
**73**	**C**	3	3	1.1	0.0	0.0	0.0	0.0	0.0	0.0	0.0	0.0	0.0	0.0	0.3	0.0	0.0	0.0	0.0	0.0	0.0	0.0	0.0	0.0	0.0
73	D	0	1	0.1	0.0	0.0	0.0	0.0	0.0	0.0	0.0	0.0	0.0	0.0	0.1	0.0	0.0	0.0	0.0	0.0	0.0	0.0	0.1	0.1	0.0
**73**	**S**	4	4	8.3	0.0	0.0	0.0	0.0	0.0	0.1	0.0	0.0	0.0	0.3	2.7	0.0	0.0	0.0	0.0	0.0	0.1	0.0	0.0	0.0	0.2
**73**	**T**	4	4	2.4	0.0	0.0	0.0	0.0	0.0	0.0	0.0	0.0	0.0	0.0	0.6	0.0	0.0	0.0	0.0	0.0	0.0	0.0	0.0	0.0	0.0
73	V	1	1	0.2	0.0	0.0	0.0	0.0	0.0	0.0	0.0	0.1	0.0	0.0	0.1	0.0	0.0	0.0	0.0	0.0	0.0	0.0	0.0	0.0	0.0
74	A	1	1	1.8	0.6	0.3	0.5	2.2	0.2	0.1	0.0	1.1	0.3	0.3	1.4	0.8	0.4	0.6	2.5	0.3	0.2	0.1	0.5	0.1	0.2
74	P	3	4	1.9	0.1	0.0	0.1	0.0	0.1	0.0	0.1	0.0	0.0	0.2	1.3	0.0	0.0	0.0	0.0	0.0	0.0	0.0	0.1	0.0	0.0
74	S	2	1	7.3	2.3	1.5	0.4	11	0.9	1.3	0.3	7.9	0.8	1.4	7.1	3.0	2.5	0.3	12	1.0	1.2	0.1	3.0	0.6	1.5
**76**	**V**	4	4	3.3	0.0	0.0	0.0	0.0	0.0	0.0	0.1	0.1	0.0	0.0	3.3	0.0	0.0	0.0	0.0	0.0	0.0	0.0	0.0	0.0	0.0
77	I	3	2	27	21	12	33	7.4	2.0	2.6	15	10	2.7	26	26	19	12	37	7.4	4.0	3.3	13	15	2.5	21
**82**	**A**	4	4	22	0.0	0.0	0.1	0.0	0.0	0.0	0.0	0.1	0.0	0.0	12	0.0	0.0	0.0	0.0	0.0	0.0	0.0	0.1	0.1	0.0
**82**	**C**	3	2	0.6	0.0	0.0	0.0	0.0	0.0	0.0	0.0	0.0	0.0	0.0	0.4	0.0	0.0	0.0	0.0	0.0	0.0	0.0	0.0	0.0	0.0
**82**	**F**	4	4	1.8	0.0	0.0	0.0	0.0	0.0	0.0	0.0	0.0	0.0	0.0	1.1	0.0	0.0	0.0	0.0	0.0	0.0	0.0	0.0	0.0	0.0
82	G	1	1	0.1	0.0	0.0	0.0	0.0	0.0	0.0	0.0	0.0	0.0	0.0	0.1	0.0	0.0	0.0	0.0	0.0	0.0	0.0	0.0	0.0	0.0
**82**	**L**	3	3	0.3	0.0	0.0	0.0	0.0	0.0	0.0	0.0	0.0	0.0	0.0	0.2	0.0	0.0	0.1	0.0	0.0	0.0	0.0	0.0	0.0	0.0
**82**	**M**	3	2	0.3	0.0	0.0	0.0	0.0	0.0	0.0	0.0	0.0	0.0	0.0	0.4	0.0	0.0	0.0	0.0	0.0	0.0	0.0	0.1	0.0	0.0
**82**	**S**	4	4	1.3	0.0	0.0	0.0	0.0	0.0	0.0	0.0	0.0	0.0	0.0	0.5	0.0	0.0	0.0	0.0	0.0	0.0	0.0	0.0	0.0	0.0
**82**	**T**	4	4	2.8	0.0	0.0	0.0	0.0	0.0	0.0	0.0	0.0	0.0	0.0	0.8	0.0	0.0	0.0	0.0	0.0	0.0	0.0	0.0	0.0	0.0
**83**	**D**	2	3	0.9	0.0	0.0	0.0	0.0	0.0	0.0	0.0	0.0	0.0	0.0	0.6	0.0	0.0	0.0	0.0	0.1	0.0	0.0	0.0	0.1	0.0
**84**	**A**	3	3	0.1	0.0	0.0	0.0	0.0	0.0	0.0	0.0	0.0	0.0	0.0	0.0	0.0	0.0	0.0	0.0	0.0	0.0	0.0	0.0	0.0	0.0
**84**	**C**	2	2	0.1	0.0	0.0	0.0	0.0	0.0	0.0	0.0	0.0	0.0	0.0	0.0	0.0	0.0	0.0	0.0	0.0	0.0	0.0	0.0	0.0	0.0
**84**	**V**	4	4	12	0.0	0.0	0.0	0.0	0.0	0.0	0.1	0.0	0.0	0.0	3.8	0.0	0.0	0.1	0.0	0.0	0.0	0.0	0.0	0.0	0.1
**85**	**V**	3	3	4.6	0.2	0.1	0.2	0.1	0.1	0.1	0.5	0.0	0.0	0.2	2.1	0.2	0.4	0.2	0.1	0.1	0.1	0.2	0.0	0.0	0.2
**88**	**D**	4	4	5.4	0.0	0.0	0.0	0.0	0.1	0.0	0.0	0.0	0.0	0.0	2.7	0.0	0.1	0.0	0.0	0.0	0.0	0.0	0.0	0.1	0.0
88	G	1	1	0.2	0.0	0.0	0.0	0.0	0.0	0.0	0.0	0.0	0.0	0.0	0.1	0.0	0.0	0.0	0.0	0.0	0.0	0.0	0.0	0.0	0.0
**88**	**S**	4	4	1.6	0.0	0.0	0.0	0.0	0.0	0.1	0.0	0.0	0.1	0.0	1.0	0.0	0.0	0.0	0.0	0.0	0.0	0.0	0.0	0.0	0.0

88	T	1	1	0.1	0.0	0.0	0.0	0.0	0.0	0.0	0.0	0.0	0.0	0.0	0.1	0.0	0.0	0.0	0.0	0.0	0.0	0.0	0.0	0.0	0.0
89	I	3	2	2.9	1.3	1.6	0.1	2.3	2.1	5.9	0.2	1.0	2.4	11	4.0	2.0	2.6	0.2	3.2	2.1	7.9	0.1	1.4	3.6	5.8
89	M	2	1	14	40	97	1.6	82	96	93	2.1	80	96	55	38	51	95	1.9	76	94	92	4.9	83	95	51
89	R	1	1	0.0	0.0	0.0	0.0	0.0	0.0	0.0	0.0	0.0	0.0	0.0	0.0	0.0	0.0	0.0	0.0	0.0	0.0	0.0	0.1	0.0	0.1
89	T	3	2	0.4	0.0	0.0	0.0	0.0	0.0	0.0	0.0	0.0	0.0	0.0	0.9	0.0	0.0	0.0	0.0	0.0	0.0	0.0	0.0	0.0	0.0
89	V	4	4	3.7	0.1	0.0	0.0	0.1	0.0	0.4	0.1	0.1	0.1	0.2	1.4	0.0	0.0	0.0	0.0	0.1	0.2	0.0	0.1	0.1	0.1
**90**	**M**	4	4	30	0.2	0.0	0.3	0.2	0.1	0.1	0.1	0.1	0.3	0.2	9.6	0.2	0.1	0.4	0.1	0.0	0.2	0.0	0.1	0.4	0.1
93	L	2	1	40	41	21	42	96	19	3.5	3.9	8.8	4.2	21	45	49	17	53	96	35	0.7	11	14	2.5	42
93	M	3	1	0.7	0.1	0.3	0.1	0.0	0.2	0.3	0.1	0.6	0.2	0.6	0.2	0.1	0.3	0.1	0.0	0.3	0.4	0.0	0.4	0.0	0.4

Abbreviation: Pos – amino acid position; AA – amino acid; List – Number of expert lists with the mutation in 2009 and 2020; %PI – percent prevalence among PI-experienced persons; %PI-Naïve – percent prevalence among PI-naïve persons.
The parenthetical numbers in the headers represent the number of persons from whom a sequence was available.
[1]40 PI-associated surveillance drug resistance mutations at 18 positions (Bennett 2009) were indicated in bold.

Table S2. List of 66 NRTI-associated mutations included in one or more of four expert 2020 lists: prevalence in RTI-naïve and NRTI-experienced persons before and after 2009.

Pos[1]	AA[1]	Lists		<2009											≥2009										
		2009	2020	%NRTI	%RTI-Naive										%NRTI	%RTI-Naive									
				(32,363)	ALL (54,910)	A (4,245)	B (30,428)	C (7,596)	01 (4,571)	02 (2,871)	D (1,279)	F (606)	G (1,003)	Other (2,361)	(24,210)	ALL (45,940)	A (2,423)	B (18,847)	C (4,811)	01 (9,495)	02 (2,382)	D (612)	F (777)	G (495)	Other (6,048)
40	F	0	1	0.6	0.0	0.0	0.0	0.0	0.0	0.0	0.0	0.0	0.0	0.0	0.1	0.0	0.0	0.0	0.0	0.0	0.0	0.0	0.0	0.0	0.0
**41**	**L**	4	4	33	0.3	0.1	0.4	0.1	0.0	0.2	0.8	0.2	0.5	0.0	15	0.2	0.5	0.3	0.1	0.1	0.3	0.5	0.3	0.2	0.1
44	A	3	1	1.7	0.0	0.0	0.0	0.0	0.0	0.0	0.1	0.0	0.0	0.0	0.6	0.0	0.0	0.0	0.0	0.0	0.0	0.0	0.0	0.0	0.0
44	D	3	1	8.7	0.3	0.8	0.2	0.1	0.0	0.1	2.2	0.5	0.2	0.3	4.3	0.1	0.5	0.1	0.2	0.0	0.1	1.5	0.4	0.6	0.1
62	V	2	2	4.1	0.7	6.9	0.1	0.1	0.1	0.1	0.0	0.3	0.1	0.1	6.9	0.5	6.5	0.1	0.1	0.2	0.0	0.0	0.0	0.0	0.3
65	E	0	3	0.1	0.0	0.0	0.0	0.0	0.0	0.1	0.0	0.2	0.0	0.0	0.1	0.0	0.0	0.0	0.0	0.0	0.0	0.3	0.0	0.0	0.1
65	N	2	4	0.1	0.0	0.0	0.0	0.0	0.0	0.0	0.0	0.0	0.0	0.0	0.2	0.0	0.0	0.0	0.0	0.0	0.1	0.0	0.0	0.0	0.0
**65**	**R**	4	4	4.0	0.0	0.1	0.0	0.1	0.0	0.0	0.0	0.0	0.0	0.0	10	0.0	0.0	0.0	0.0	0.0	0.0	0.0	0.0	0.0	0.0
67	del	2	2	0.1	0.0	0.0	0.0	0.0	0.0	0.0	0.0	0.0	0.0	0.0	0.0	0.0	0.0	0.0	0.0	0.0	0.0	0.0	0.0	0.0	0.0
**67**	**E**	2	1	0.6	0.0	0.0	0.0	0.0	0.0	0.1	0.0	0.0	0.1	0.1	0.2	0.0	0.0	0.0	0.0	0.0	0.0	0.2	0.0	0.0	0.0
**67**	**G**	3	2	2.4	0.0	0.0	0.0	0.0	0.0	0.0	0.0	0.0	0.0	0.1	2.3	0.0	0.0	0.0	0.1	0.0	0.0	0.2	0.0	0.0	0.0
67	H	1	1	0.2	0.0	0.0	0.0	0.0	0.0	0.0	0.0	0.0	0.0	0.0	0.1	0.0	0.0	0.0	0.0	0.0	0.0	0.0	0.0	0.0	0.0
**67**	**N**	4	4	30	0.1	0.1	0.0	0.0	0.0	0.2	0.1	0.0	0.0	0.0	16	0.0	0.0	0.0	0.0	0.0	0.0	0.0	0.1	0.0	0.1
67	S	1	1	0.3	0.0	0.0	0.0	0.0	0.0	0.0	0.0	0.0	0.0	0.0	0.2	0.0	0.0	0.0	0.0	0.0	0.0	0.0	0.0	0.0	0.0
67	T	1	1	0.2	0.0	0.0	0.0	0.0	0.0	0.0	0.0	0.0	0.0	0.0	0.2	0.0	0.0	0.0	0.0	0.0	0.0	0.0	0.0	0.0	0.0
68	del	0	1	0.0	0.0	0.0	0.0	0.0	0.0	0.0	0.0	0.0	0.0	0.0	0.0	0.0	0.0	0.0	0.0	0.0	0.0	0.0	0.0	0.0	0.0
69	A	3	1	1.1	0.2	0.0	0.3	0.2	0.2	0.1	0.2	0.5	0.1	0.1	0.9	0.3	0.2	0.4	0.3	0.2	0.3	0.0	0.4	0.0	0.1
**69**	**D**	3	3	6.9	0.1	0.0	0.1	0.1	0.0	0.0	0.2	0.0	0.0	0.0	3.1	0.1	0.0	0.1	0.1	0.1	0.1	0.0	0.0	0.0	0.0
69	del	1	1	0.1	0.0	0.0	0.0	0.0	0.0	0.0	0.0	0.0	0.0	0.0	0.3	0.0	0.0	0.0	0.0	0.0	0.0	0.0	0.0	0.0	0.0
69	G	2	1	0.3	0.0	0.0	0.0	0.0	0.0	0.0	0.0	0.0	0.0	0.0	0.1	0.0	0.0	0.0	0.0	0.0	0.0	0.0	0.0	0.0	0.0
**69**	**ins**	4	4	1.0	0.0	0.0	0.0	0.0	0.0	0.0	0.0	0.0	0.0	0.0	0.2	0.0	0.0	0.0	0.0	0.0	0.0	0.0	0.0	0.0	0.0
69	N	3	2	6.5	0.6	0.3	0.6	0.3	2.1	0.4	0.2	0.3	0.0	0.1	5.1	0.8	0.5	0.7	0.2	1.7	0.5	0.7	0.5	0.8	0.3
69	S	2	1	1.1	0.8	0.9	0.8	0.4	0.7	0.4	0.8	0.7	0.5	0.7	1.0	1.2	1.4	0.6	0.4	1.3	0.8	0.2	0.4	1.8	0.6
70	del	0	1	0.0	0.0	0.0	0.0	0.0	0.0	0.0	0.0	0.0	0.0	0.0	0.0	0.0	0.0	0.0	0.0	0.0	0.0	0.0	0.0	0.0	0.0
**70**	**E**	4	4	0.6	0.0	0.0	0.0	0.0	0.0	0.1	0.2	0.0	0.0	0.0	2.6	0.0	0.0	0.0	0.0	0.0	0.0	0.0	0.0	0.0	0.0
70	G	2	2	0.5	0.0	0.0	0.0	0.0	0.0	0.0	0.0	0.0	0.0	0.0	0.3	0.0	0.0	0.0	0.0	0.0	0.0	0.0	0.0	0.0	0.0
70	N	1	1	0.3	0.0	0.0	0.0	0.0	0.0	0.1	0.0	0.0	0.0	0.1	0.4	0.0	0.0	0.1	0.0	0.0	0.0	0.0	0.1	0.0	0.1
70	Q	0	1	0.2	0.0	0.0	0.0	0.0	0.0	0.0	0.0	0.0	0.0	0.0	0.6	0.0	0.0	0.0	0.0	0.0	0.0	0.0	0.0	0.0	0.0
**70**	**R**	4	4	21	0.1	0.0	0.1	0.0	0.1	0.0	0.1	0.2	0.0	0.0	12	0.0	0.1	0.1	0.0	0.0	0.0	0.0	0.0	0.0	0.1
70	S	1	1	0.1	0.0	0.0	0.0	0.0	0.0	0.0	0.0	0.0	0.0	0.0	0.1	0.0	0.0	0.0	0.0	0.0	0.0	0.0	0.0	0.0	0.0

70	T	1	1	0.3	0.0	0.0	0.0	0.0	0.1	0.0	0.0	0.2	0.0	0.0	0.7	0.0	0.0	0.0	0.0	0.1	0.0	0.0	0.0	0.0	0.0
**74**	**I**	3	3	4.6	0.0	0.0	0.0	0.0	0.1	0.0	0.0	0.0	0.0	0.0	3.7	0.0	0.0	0.0	0.0	0.1	0.0	0.0	0.0	0.0	0.1
**74**	**V**	4	4	8.6	0.0	0.0	0.0	0.0	0.0	0.0	0.0	0.0	0.1	0.0	3.9	0.0	0.0	0.0	0.0	0.0	0.0	0.0	0.0	0.0	0.1
**75**	**A**	3	2	0.8	0.0	0.0	0.0	0.0	0.0	0.0	0.0	0.0	0.0	0.0	0.2	0.0	0.0	0.0	0.0	0.0	0.0	0.0	0.0	0.0	0.1
75	I	2	3	3.2	0.0	0.0	0.0	0.0	0.0	0.1	1.0	0.0	0.1	0.0	2.7	0.0	0.0	0.0	0.0	0.0	0.0	0.2	0.0	0.0	0.1
**75**	**M**	3	2	3.6	0.0	0.0	0.0	0.0	0.2	0.0	0.0	0.0	0.1	0.0	3.5	0.1	0.0	0.1	0.0	0.1	0.0	0.0	0.0	0.0	0.0
**75**	**S**	3	2	0.3	0.0	0.0	0.0	0.0	0.0	0.0	0.0	0.0	0.0	0.1	0.1	0.0	0.0	0.0	0.0	0.0	0.0	0.0	0.0	0.0	0.0
**75**	**T**	3	2	1.5	0.0	0.0	0.0	0.0	0.0	0.0	0.0	0.0	0.0	0.0	0.5	0.0	0.0	0.0	0.0	0.0	0.0	0.0	0.0	0.0	0.0
**77**	**L**	2	3	1.9	0.2	0.0	0.2	0.0	0.1	0.1	0.0	0.2	0.0	0.0	1.2	0.1	0.1	0.1	0.0	0.0	0.0	0.0	0.0	0.0	0.0
**115**	**F**	4	4	2.0	0.0	0.0	0.0	0.0	0.0	0.1	0.0	0.0	0.0	0.0	4.5	0.0	0.0	0.0	0.0	0.0	0.0	0.0	0.0	0.2	0.0
**116**	**Y**	2	2	2.2	0.0	0.0	0.0	0.0	0.0	0.0	0.0	0.0	0.0	0.0	1.0	0.0	0.0	0.0	0.0	0.0	0.0	0.0	0.0	0.0	0.0
118	I	2	1	18	2.7	2.4	3.2	2.6	2.1	0.9	3.5	2.6	2.0	2.2	8.5	4.4	3.0	6.1	2.8	5.1	1.1	2.0	3.5	2.8	3.8
151	L	1	1	0.2	0.0	0.0	0.0	0.0	0.0	0.0	0.0	0.0	0.0	0.0	0.1	0.0	0.0	0.0	0.0	0.0	0.0	0.0	0.0	0.0	0.0
**151**	**M**	4	4	2.9	0.0	0.0	0.0	0.0	0.0	0.0	0.0	0.0	0.0	0.0	1.2	0.0	0.0	0.0	0.0	0.0	0.0	0.0	0.0	0.0	0.0
**184**	**I**	4	4	2.3	0.0	0.0	0.0	0.0	0.1	0.0	0.1	0.0	0.0	0.0	3.3	0.1	0.0	0.1	0.1	0.0	0.1	0.0	0.0	0.2	0.0
**184**	**V**	4	4	55	0.2	0.2	0.3	0.1	0.2	0.1	0.2	0.2	0.2	0.1	61	0.1	0.0	0.2	0.1	0.1	0.2	0.2	0.0	0.2	0.2
**210**	**W**	4	4	21	0.1	0.1	0.1	0.0	0.0	0.1	0.0	0.0	0.0	0.0	7.7	0.1	0.1	0.1	0.1	0.1	0.1	0.0	0.1	0.0	0.2
215	A	2	3	0.3	0.1	0.1	0.2	0.0	0.0	0.1	0.1	0.0	0.2	0.1	0.3	0.1	0.0	0.1	0.0	0.1	0.0	0.2	0.0	0.2	0.0
**215**	**C**	3	3	0.9	0.1	0.0	0.1	0.0	0.0	0.0	0.0	0.0	0.0	0.0	0.4	0.1	0.0	0.2	0.0	0.0	0.0	0.0	0.0	0.0	0.0
**215**	**D**	3	3	0.8	0.3	0.0	0.5	0.0	0.0	0.0	0.1	0.0	0.0	0.0	0.4	0.2	0.0	0.4	0.0	0.0	0.1	0.2	0.0	0.0	0.0
**215**	**E**	3	3	0.2	0.1	0.0	0.2	0.0	0.0	0.0	0.0	0.0	0.1	0.0	0.1	0.2	0.0	0.4	0.0	0.0	0.0	0.0	0.0	0.0	0.0
**215**	**F**	4	4	12	0.0	0.0	0.0	0.0	0.0	0.0	0.0	0.0	0.0	0.0	8.3	0.0	0.0	0.0	0.0	0.0	0.0	0.0	0.0	0.0	0.0
215	G	2	2	0.1	0.0	0.0	0.0	0.0	0.0	0.0	0.0	0.0	0.0	0.0	0.0	0.0	0.0	0.0	0.0	0.0	0.0	0.0	0.0	0.0	0.0
215	H	2	2	0.1	0.0	0.0	0.0	0.0	0.0	0.0	0.0	0.0	0.0	0.0	0.0	0.0	0.0	0.0	0.0	0.0	0.0	0.0	0.0	0.0	0.0
**215**	**I**	3	3	2.2	0.0	0.1	0.0	0.0	0.0	0.0	0.1	0.0	0.0	0.1	2.0	0.0	0.0	0.0	0.0	0.0	0.0	0.0	0.1	0.0	0.1
215	L	2	3	0.2	0.1	0.0	0.1	0.0	0.0	0.0	0.0	0.0	0.0	0.1	0.2	0.1	0.0	0.1	0.0	0.0	0.0	0.0	0.0	0.0	0.0
215	N	2	3	1.3	0.0	0.0	0.0	0.0	0.0	0.0	0.0	0.0	0.0	0.0	0.7	0.0	0.0	0.0	0.0	0.0	0.0	0.0	0.0	0.0	0.0
**215**	**S**	3	3	2.5	0.4	0.1	0.6	0.1	0.1	0.1	0.0	0.5	0.2	0.3	1.8	0.3	0.0	0.6	0.1	0.2	0.1	0.5	0.1	0.2	0.1
**215**	**V**	3	3	0.9	0.0	0.0	0.0	0.0	0.0	0.0	0.0	0.0	0.0	0.0	0.5	0.0	0.0	0.0	0.0	0.0	0.0	0.0	0.0	0.0	0.0
**215**	**Y**	4	4	31	0.0	0.0	0.0	0.0	0.0	0.0	0.0	0.0	0.0	0.0	13	0.0	0.0	0.0	0.0	0.0	0.0	0.0	0.0	0.0	0.0
**219**	**E**	4	4	6.8	0.0	0.0	0.0	0.0	0.0	0.0	0.0	0.0	0.2	0.1	6.1	0.0	0.0	0.0	0.0	0.0	0.0	0.0	0.0	0.0	0.0
219	H	1	1	0.3	0.0	0.0	0.0	0.0	0.0	0.0	0.0	0.0	0.0	0.0	0.1	0.0	0.0	0.0	0.0	0.0	0.0	0.0	0.0	0.0	0.0
**219**	**N**	3	2	3.4	0.0	0.1	0.0	0.0	0.1	0.1	0.1	0.2	0.0	0.3	1.4	0.0	0.0	0.0	0.0	0.0	0.0	0.0	0.0	0.2	0.1
**219**	**Q**	4	4	13	0.1	0.2	0.1	0.0	0.1	0.2	0.2	0.2	0.1	0.0	6.9	0.1	0.1	0.1	0.1	0.1	0.1	0.3	0.0	0.0	0.0
**219**	**R**	3	2	2.7	0.1	0.1	0.1	0.1	0.1	0.1	0.0	0.0	0.0	0.3	1.3	0.0	0.0	0.1	0.0	0.0	0.0	0.2	0.0	0.0	0.0
219	W	1	1	0.3	0.0	0.0	0.0	0.0	0.0	0.0	0.0	0.0	0.0	0.0	0.2	0.0	0.0	0.0	0.0	0.0	0.0	0.0	0.0	0.0	0.0

Abbreviation: Pos – amino acid position; AA – amino acid; List – Number of expert lists with the mutation in 2009 and 2020; %NRTI – percent prevalence among NRTI-experienced persons; %RTI-Naïve – percent prevalence among RTI-naïve persons.
The parenthetical numbers in the headers represent the number of persons from whom a sequence was available
[1]34 NRTI-associated surveillance drug resistance mutations at 15 positions (Bennett 2009) were indicated in bold.

Table S3. List of 69 NNRTI-associated mutations included in one or more of four expert 2020 lists: prevalence in RTI-naïve and NNRTI-experienced persons before and after 2009.

Pos[1]	AA[1]	Lists		<2009											≥2009										
		2009	2020	%NNRTI	%RTI-Naive										%NNRTI	%RTI-Naive									
				(27,755)	ALL (54,910)	A (4,245)	B (30,428)	C (7,596)	01 (4,571)	02 (2,871)	D (1,279)	F (606)	G (1,003)	Other (2,361)	(22,219)	ALL (45,940)	A (2,423)	B (18,847)	C (4,811)	01 (9,495)	02 (2,382)	D (612)	F (777)	G (495)	Other (6,048)
90	I	2	3	5.9	1.9	1.4	2.1	1.0	0.5	6.8	2.6	0.2	1.2	0.8	8.6	1.6	1.9	1.7	1.1	0.5	7.2	1.6	0.0	3.6	0.5
98	G	4	4	6.4	0.2	0.1	0.1	0.8	0.2	0.1	0.1	0.5	0.3	0.0	8.3	0.2	0.1	0.2	0.6	0.1	0.3	0.2	0.9	1.0	0.2
**100**	**I**	4	4	4.5	0.0	0.0	0.0	0.1	0.0	0.0	0.2	0.0	0.0	0.0	3.5	0.0	0.0	0.0	0.0	0.0	0.0	0.0	0.0	0.0	0.0
100	V	1	1	0.1	0.0	0.0	0.1	0.0	0.0	0.0	0.0	0.0	0.0	0.0	0.1	0.0	0.0	0.0	0.0	0.0	0.0	0.0	0.0	0.0	0.1
**101**	**E**	4	4	7.2	0.1	0.1	0.2	0.1	0.0	0.1	0.4	0.0	0.1	0.0	9.3	0.2	0.2	0.2	0.3	0.1	0.1	0.2	0.0	0.8	0.1
101	H	1	3	1.3	0.0	0.0	0.0	0.0	0.0	0.0	0.0	0.0	0.0	0.0	1.3	0.0	0.0	0.0	0.0	0.0	0.0	0.0	0.0	0.0	0.0
101	I	0	1	0.1	0.0	0.0	0.0	0.0	0.0	0.0	0.0	0.2	0.0	0.0	0.1	0.0	0.0	0.0	0.0	0.0	0.0	0.0	0.0	0.0	0.0
101	N	2	1	0.5	0.0	0.0	0.0	0.0	0.0	0.1	0.0	0.0	0.0	0.0	0.7	0.0	0.1	0.0	0.0	0.0	0.0	0.0	0.0	0.2	0.0
**101**	**P**	4	4	1.7	0.0	0.0	0.0	0.0	0.0	0.0	0.0	0.0	0.0	0.0	1.4	0.0	0.0	0.0	0.0	0.0	0.0	0.0	0.0	0.0	0.0
101	Q	2	1	4.9	0.5	0.4	0.6	0.5	0.3	0.6	0.2	0.2	0.7	0.1	3.9	0.7	0.9	0.9	0.6	0.4	0.8	0.5	0.5	0.2	0.9
101	R	2	2	1.6	1.0	0.3	1.2	0.9	0.7	0.3	1.1	0.8	0.6	2.0	1.6	1.1	0.7	1.2	1.1	0.9	0.6	0.5	0.6	0.0	1.0
101	T	0	1	0.4	0.0	0.0	0.0	0.0	0.0	0.0	0.0	0.0	0.0	0.0	0.2	0.0	0.0	0.0	0.0	0.0	0.0	0.0	0.0	0.0	0.0
103	H	2	3	0.1	0.0	0.0	0.0	0.0	0.0	0.0	0.0	0.0	0.0	0.0	0.0	0.0	0.0	0.0	0.0	0.0	0.0	0.0	0.0	0.0	0.0
**103**	**N**	4	4	36	1.3	0.5	1.8	1.1	0.2	0.7	0.9	1.5	0.9	0.7	36	1.6	2.1	2.3	1.5	0.4	2.4	2.3	1.5	3.0	1.3
103	R	2	2	2.4	1.9	0.2	2.9	1.2	0.8	0.2	0.4	0.3	0.2	0.4	2.8	2.0	0.3	3.1	1.2	2.1	0.7	0.3	1.0	0.6	1.6
**103**	**S**	3	4	2.0	0.0	0.0	0.0	0.1	0.0	0.0	0.0	0.2	0.0	0.0	2.3	0.1	0.2	0.1	0.0	0.0	0.0	0.2	0.0	0.0	0.1
103	T	3	3	0.2	0.0	0.0	0.0	0.0	0.0	0.0	0.0	0.0	0.0	0.0	0.1	0.0	0.0	0.0	0.0	0.0	0.0	0.0	0.0	0.0	0.0
**106**	**A**	4	4	1.8	0.0	0.0	0.0	0.0	0.0	0.0	0.0	0.0	0.1	0.0	1.4	0.0	0.0	0.0	0.0	0.0	0.0	0.0	0.0	0.0	0.1
106	I	4	4	3.9	1.9	1.3	2.2	0.0	4.8	0.5	1.6	4.6	1.1	2.0	3.5	3.0	1.7	4.5	0.2	2.9	1.4	2.1	16	2.2	1.1
**106**	**M**	4	4	2.6	0.0	0.0	0.0	0.0	0.0	0.0	0.0	0.0	0.0	0.0	9.1	0.1	0.0	0.0	0.2	0.1	0.0	0.0	0.0	0.2	0.0
106	T	0	1	0.0	0.0	0.0	0.0	0.0	0.0	0.0	0.0	0.0	0.0	0.0	0.0	0.0	0.0	0.0	0.0	0.0	0.0	0.0	0.0	0.0	0.0
108	I	3	3	8.6	0.5	0.7	0.5	0.2	0.2	0.7	0.3	0.3	0.1	0.4	9.4	0.4	0.3	0.5	0.2	0.2	0.9	0.3	0.3	0.2	0.1
138	A	0	4	3.1	2.5	3.2	1.9	6.0	0.4	2.7	3.0	3.3	2.2	1.6	4.9	2.2	6.2	1.9	5.4	0.3	2.7	2.1	1.7	1.8	0.6
138	G	0	4	0.7	0.2	0.2	0.2	0.3	0.4	0.1	0.5	2.0	0.1	0.1	1.1	0.4	0.2	0.3	0.3	0.2	0.3	0.3	0.5	0.2	1.6
138	K	2	4	0.4	0.1	0.0	0.1	0.2	0.0	0.1	0.3	0.0	0.0	0.0	0.7	0.1	0.0	0.1	0.1	0.1	0.1	0.3	0.1	0.2	0.2
138	Q	0	4	1.0	0.0	0.1	0.0	0.0	0.0	0.0	0.1	0.2	0.0	0.0	1.6	0.1	0.1	0.1	0.0	0.0	0.0	0.0	0.0	0.2	0.0
138	R	0	4	0.0	0.0	0.0	0.0	0.0	0.0	0.1	0.0	0.0	0.0	0.0	0.1	0.0	0.0	0.0	0.0	0.0	0.0	0.0	0.0	0.0	0.0
138	S	0	2	0.0	0.0	0.0	0.0	0.3	0.0	0.0	0.0	0.0	0.0	0.0	0.1	0.0	0.1	0.0	0.1	0.0	0.0	0.0	0.0	0.0	0.0
179	D	4	4	2.2	1.6	0.2	2.1	1.1	2.2	0.0	0.2	3.8	0.0	0.7	3.3	2.4	0.5	3.1	1.8	3.3	0.0	0.5	0.8	0.0	1.9
179	E	2	2	1.2	0.6	0.0	0.6	0.1	0.2	0.8	0.4	0.0	4.3	0.5	1.8	1.5	0.0	1.4	0.3	0.7	1.0	0.2	0.3	3.4	15

**179**	**F**	4	3	0.3	0.0	0.0	0.0	0.0	0.0	0.0	0.0	0.0	0.0	0.1	0.2	0.0	0.0	0.0	0.0	0.0	0.0	0.0	0.0	0.0	0.0
179	I	2	1	13	8.9	54	4.3	3.1	16.2	3.9	7.0	5.3	0.4	6.6	16	10	51	5.4	3.7	20	5.3	6.9	17	0.8	6.1
179	L	0	4	0.2	0.0	0.0	0.0	0.0	0.0	0.0	0.0	0.0	0.0	0.0	0.3	0.0	0.0	0.0	0.0	0.0	0.1	0.0	0.0	0.0	0.0
179	M	1	1	0.1	0.0	0.0	0.0	0.0	0.0	0.2	0.0	0.0	0.0	0.0	0.3	0.0	0.0	0.0	0.0	0.0	0.1	0.0	0.1	0.0	0.0
179	T	1	3	0.5	0.3	1.6	0.1	0.0	1.1	0.1	1.0	0.2	0.0	0.4	0.9	0.8	1.7	0.2	0.1	2.9	0.2	1.5	0.1	0.0	0.9
**181**	**C**	4	4	18	0.2	0.1	0.1	0.3	0.2	0.1	0.2	0.0	0.2	0.0	18	0.2	0.2	0.1	0.2	0.1	0.3	0.7	0.1	0.4	0.3
181	F	0	1	0.2	0.0	0.0	0.0	0.0	0.0	0.0	0.0	0.0	0.0	0.0	0.2	0.0	0.1	0.0	0.1	0.0	0.0	0.0	0.0	0.0	0.0
181	G	0	1	0.1	0.0	0.0	0.0	0.0	0.0	0.0	0.0	0.0	0.0	0.0	0.1	0.0	0.0	0.0	0.0	0.0	0.0	0.0	0.0	0.0	0.0
**181**	**I**	4	4	0.8	0.0	0.0	0.0	0.0	0.0	0.0	0.0	0.0	0.0	0.0	0.6	0.0	0.1	0.0	0.0	0.0	0.0	0.0	0.0	0.0	0.0
181	S	1	1	0.1	0.0	0.0	0.0	0.0	0.0	0.0	0.0	0.0	0.0	0.0	0.1	0.0	0.0	0.0	0.0	0.0	0.0	0.0	0.1	0.0	0.0
**181**	**V**	4	4	0.5	0.0	0.0	0.0	0.0	0.0	0.0	0.0	0.0	0.0	0.0	0.7	0.0	0.0	0.0	0.0	0.0	0.0	0.0	0.0	0.0	0.0
**188**	**C**	4	4	0.7	0.0	0.0	0.0	0.0	0.0	0.0	0.0	0.0	0.0	0.0	1.0	0.0	0.1	0.0	0.0	0.0	0.0	0.0	0.0	0.0	0.0
188	F	1	2	0.6	0.0	0.0	0.0	0.0	0.0	0.0	0.0	0.0	0.0	0.0	0.6	0.0	0.0	0.0	0.0	0.0	0.0	0.0	0.0	0.0	0.1
**188**	**H**	4	4	0.8	0.0	0.0	0.0	0.0	0.0	0.0	0.1	0.0	0.0	0.1	0.7	0.0	0.0	0.0	0.0	0.0	0.0	0.0	0.0	0.0	0.0
**188**	**L**	4	4	4.5	0.0	0.0	0.1	0.0	0.0	0.0	0.0	0.0	0.3	0.0	4.4	0.1	0.0	0.1	0.0	0.0	0.0	0.5	0.0	0.0	0.1
189	I	0	1	2.6	1.2	0.9	1.2	0.6	0.7	3.3	1.8	0.8	2.7	0.7	2.3	1.3	1.2	1.3	0.9	1.3	2.5	1.6	1.0	1.8	1.0
**190**	**A**	4	4	15	0.2	0.0	0.2	0.1	0.1	0.1	0.2	0.0	0.0	0.0	15	0.2	0.3	0.2	0.3	0.1	0.1	4.7	0.1	0.0	0.1
190	C	3	3	0.1	0.0	0.0	0.0	0.0	0.0	0.0	0.0	0.0	0.0	0.0	0.1	0.0	0.0	0.0	0.0	0.0	0.0	0.0	0.0	0.0	0.0
**190**	**E**	3	4	0.5	0.0	0.0	0.0	0.0	0.1	0.0	0.0	0.0	0.0	0.0	0.6	0.1	0.0	0.0	0.1	0.1	0.0	0.0	0.0	0.0	0.1
190	Q	3	3	0.3	0.0	0.0	0.0	0.0	0.0	0.0	0.0	0.0	0.0	0.0	0.4	0.0	0.0	0.0	0.0	0.0	0.0	0.0	0.0	0.0	0.0
**190**	**S**	4	4	2.7	0.0	0.0	0.0	0.0	0.0	0.0	0.0	0.0	0.0	0.0	3.3	0.0	0.0	0.0	0.0	0.0	0.0	0.2	0.0	0.0	0.0
190	T	3	3	0.1	0.0	0.0	0.0	0.0	0.0	0.0	0.0	0.0	0.0	0.0	0.1	0.0	0.0	0.0	0.0	0.0	0.0	0.0	0.0	0.0	0.0
190	V	3	3	0.0	0.0	0.0	0.0	0.0	0.0	0.0	0.1	0.0	0.0	0.0	0.0	0.0	0.0	0.0	0.0	0.0	0.0	0.0	0.0	0.0	0.0
221	Y	1	4	6.5	0.1	0.1	0.1	0.1	0.0	0.1	0.2	0.2	0.0	0.1	8.1	0.1	0.1	0.1	0.1	0.1	0.0	0.2	0.0	0.4	0.1
**225**	**H**	4	4	4.3	0.0	0.0	0.0	0.0	0.0	0.1	0.0	0.0	0.0	0.0	7.4	0.0	0.0	0.0	0.0	0.0	0.0	0.0	0.0	0.0	0.0
227	C	2	4	0.0	0.0	0.0	0.0	0.0	0.0	0.0	0.0	0.0	0.0	0.0	0.1	0.0	0.0	0.0	0.0	0.0	0.0	0.0	0.0	0.0	0.0
227	I	0	2	0.0	0.0	0.0	0.1	0.0	0.0	0.1	0.0	0.0	0.0	0.0	0.0	0.0	0.0	0.0	0.0	0.0	0.0	0.0	0.0	0.0	0.0
227	L	2	3	2.2	0.1	0.0	0.1	0.0	0.1	0.0	0.0	0.2	0.0	0.0	3.5	0.0	0.0	0.0	0.0	0.0	0.0	0.0	0.0	0.0	0.0
227	R	0	1	0.0	0.0	0.0	0.0	0.0	0.0	0.0	0.0	0.0	0.0	0.0	0.0	0.0	0.0	0.0	0.0	0.0	0.0	0.0	0.0	0.0	0.0
227	V	0	1	0.0	0.0	0.0	0.0	0.0	0.0	0.0	0.0	0.2	0.0	0.0	0.0	0.0	0.0	0.0	0.0	0.0	0.0	0.0	0.0	0.0	0.0
230	I	0	4	0.0	0.0	0.0	0.0	0.0	0.0	0.0	0.0	0.0	0.1	0.0	0.1	0.0	0.0	0.0	0.0	0.0	0.0	0.0	0.0	0.0	0.0
**230**	**L**	3	4	1.5	0.0	0.0	0.0	0.0	0.0	0.0	0.0	0.0	0.0	0.0	2.6	0.0	0.0	0.0	0.0	0.0	0.0	0.0	0.0	0.0	0.1
230	V	0	1	0.1	0.0	0.0	0.0	0.0	0.1	0.1	0.0	0.0	0.0	0.0	0.0	0.0	0.0	0.0	0.0	0.0	0.0	0.0	0.0	0.0	0.0
234	I	1	3	0.3	0.0	0.1	0.0	0.0	0.0	0.1	0.1	0.0	0.0	0.0	0.4	0.0	0.0	0.0	0.0	0.0	0.0	0.0	0.0	0.0	0.1
236	L	2	2	0.1	0.0	0.0	0.0	0.0	0.0	0.2	0.0	0.2	0.0	0.2	0.2	0.0	0.0	0.1	0.0	0.0	0.0	0.0	0.0	0.0	0.0
238	N	1	2	0.6	0.0	0.0	0.1	0.0	0.0	0.1	0.1	0.2	0.1	0.0	0.4	0.1	0.0	0.1	0.0	0.0	0.2	0.0	0.0	0.0	0.0
238	T	2	2	2.7	0.1	0.1	0.1	0.0	0.0	0.0	0.0	0.2	0.0	0.0	3.0	0.0	0.0	0.1	0.0	0.0	0.1	0.2	0.0	0.0	0.0

318	F	2	2	1.2	0.1	0.1	0.1	0.1	0.0	0.2	0.0	0.8	0.0	0.0	1.3	0.2	0.0	0.1	0.2	0.1	0.8	0.0	0.0	1.9	0.0
348	I	0	1	14	0.2	0.3	0.2	0.4	0.0	0.2	0.0	0.0	0.0	0.0	7.0	0.0	0.0	0.0	0.0	0.1	0.0	0.0	0.0	0.0	0.0

Abbreviation: Pos – amino acid position; AA – amino acid; List – Number of expert lists with the mutation in 2009 and 2020; %NNRTI – percent prevalence among NNRTI-experienced persons; %RTI-Naïve – percent prevalence among RTI-naïve persons.
The parenthetical numbers in the headers represent the number of persons from whom a sequence was available
[1]19 NNRTI-associated surveillance drug resistance mutations at 10 positions (Bennett 2009) were indicated in bold.

Table S4. Number of sequences according to treatment and subtype

Subtype	#Protease sequences		#RT sequences	
	PI-Naïve (n=115,880)	PI-Experienced (n=32,800)	RTI-Naïve (n=100,850)	RTI-Experienced (n=56,573)
All	115,880	328,00	100,850	56,573
A	7,525	1,532	6,668	3,873
B	54,567	22,236	49,275	29,132
C	19,129	3,617	12,407	12,109
CRF01_AE	15,454	696	14,066	3,061
CRF02_AG	7,359	366	5,253	2,289
D	2,388	390	1,891	983
F	1,520	1,529	1,383	1,611
G	3461	401	1,498	1,548
CRF06_cpx	393	7	1,134	356
CRF07_BC	651	9	3,311	16
CRF08_BC	225	29	814	29
CRF12_BF	411	1,164	267	673
CRF18_cpx	224	40	104	55
CRF35_AD	430	30	241	16
CRF45_cpx	320	49	64	48
CRF63_02A1	128	77	127	86
Other	1,695	628	2,347	688

Table S5. Recently published transmitted drug resistance (TDR) studies containing sequences from >1,000 ART-naïve persons: TDR prevalence using 2009 surveillance drug resistance mutation (SDRM) list and expanded SDRM list*

Author-year	PMID	Country	Median sample year	#Pts	%TDR using 2009 SDRM list				%TDR using expanded SDRM list			
					Overall	PI	NRTI	NNRTI	Overall	PI	NRTI	NNRTI
Meixenberger14	24920768	Germany	2008	2,203	12.1	2.3	7.4	3.6	12.8	2.8	7.4	3.9
Li15	26121491	China	2012	1,265	3.2	2.1	.7	.7	3.8	2.4	.9	.9
Li15	26577039	China	2010	1,205	3.2	1.7	1.1	.8	3.7	1.9	1.1	1.1
Pouran Yousef16	27400403	Germany	2007	1,159	11.4	2.9	6.2	3.5	12.0	3.2	6.2	4.0
Zhao16	27352965	China	2011	1,316	2.0	.7	1.0	.5	2.1	.8	1.0	.5
Hattori16	26428230	Japan	2010	2,148	8.9	3.4	4.5	1.5	9.5	3.8	4.6	1.7
Hachiya17		Japan	2014	2,132	8.7	2.5	5.4	1.2	9.6	2.7	5.5	1.8
Derache18	30321314	South Africa	2014	1,148	8.1	.3	.7	7.7	8.4	.3	.7	7.8
Arruda18	29504269	Brazil	2015	1,566	10.7	2.3	4.1	5.8	11.4	2.7	4.2	6.2
Rhee18	29846534	U.S.	2011	3,292	14.2	3.3	5.2	8.2	14.7	3.6	5.3	8.4
Machnowska19	30650082	Germany	2012	2,052	10.3	2.9	6.0	3.5	11.3	3.3	6.0	4.0

*Expanded SDRM list include 2009 SDRMs, six NRTI-associated DRMs (K65N, T69deletion, K70G/N/Q/T), six NNRTI-associated DRMs (E138K/Q, V179L, H221Y, F227C/L) and three PI-associated DRMs (L10F, T74P, L89V).

Table S6. NRTI-associated SDRMs and candidate SDRMs: Comparisons of prevalence in RTI-naïve and NRTI-experienced persons before and after 2009 using Fisher's exact test.

Pos	AA	SDRM	RTI-Naive					NRTI-Experienced				
			% in <2009 (n=54,910)	% in ≥2009 (n=45,940)	OR	p	Adjusted p	% in <2009 (n=32,363)	% in ≥2009 (n=24,210)	OR	p	Adjusted p
41	L	SDRM	0.003	0.002	0.62	5.9E-05	**2.0E-03**	0.330	0.148	0.35	0.0E+00	**0.0E+00**
65	R	SDRM	0.000	0.000	0.85	4.3E-01	1.0E+00	0.040	0.104	2.80	3.0E-198	**8.9E-197**
67	E	SDRM	0.000	0.000	0.75	3.0E-01	1.0E+00	0.006	0.002	0.37	1.1E-11	**1.2E-10**
67	G	SDRM	0.000	0.000	0.50	6.9E-02	1.0E+00	0.024	0.023	0.93	9.3E-02	3.7E-01
67	N	SDRM	0.001	0.000	0.52	2.7E-02	6.9E-01	0.302	0.157	0.43	0.0E+00	**0.0E+00**
69	D	SDRM	0.001	0.001	1.17	2.8E-01	1.0E+00	0.069	0.031	0.43	8.9E-95	**2.3E-93**
69	ins	SDRM	0.000	0.000	Inf	4.6E-01	1.0E+00	0.010	0.002	0.21	1.5E-33	**2.9E-32**
70	E	SDRM	0.000	0.000	0.64	2.4E-01	1.0E+00	0.006	0.026	4.04	3.6E-79	**9.0E-78**
70	R	SDRM	0.001	0.000	0.52	8.3E-03	2.5E-01	0.206	0.124	0.54	1.6E-151	**4.6E-150**
74	I	SDRM	0.000	0.000	1.02	5.5E-01	1.0E+00	0.046	0.037	0.79	2.6E-08	**2.6E-07**
74	V	SDRM	0.000	0.000	0.40	2.1E-01	1.0E+00	0.086	0.039	0.43	5.0E-115	**1.4E-113**
75	A	SDRM	0.000	0.000	0.60	3.5E-01	1.0E+00	0.008	0.002	0.32	4.4E-18	**6.5E-17**
75	M	SDRM	0.000	0.001	1.62	8.7E-02	1.0E+00	0.036	0.035	0.96	2.0E-01	3.7E-01
75	S	SDRM	0.000	0.000	0.00	5.4E-01	1.0E+00	0.003	0.001	0.47	8.3E-05	**5.0E-04**
75	T	SDRM	0.000	0.000	0.00	1.0E+00	1.0E+00	0.015	0.005	0.32	2.4E-33	**4.1E-32**
77	L	SDRM	0.002	0.001	0.42	1.3E-05	**4.6E-04**	0.019	0.012	0.62	6.8E-12	**8.1E-11**
115	F	SDRM	0.000	0.000	0.20	9.7E-02	1.0E+00	0.020	0.045	2.27	3.0E-62	**7.3E-61**
116	Y	SDRM	0.000	0.000	0.00	8.8E-02	1.0E+00	0.022	0.010	0.43	1.4E-32	**2.3E-31**
151	M	SDRM	0.000	0.000	0.00	1.0E+00	1.0E+00	0.029	0.012	0.43	4.8E-42	**9.6E-41**
184	I	SDRM	0.000	0.001	1.91	3.3E-02	8.2E-01	0.023	0.033	1.45	4.5E-13	**5.8E-12**
184	V	SDRM	0.002	0.001	0.70	1.0E-02	3.0E-01	0.545	0.606	1.28	9.2E-48	**2.0E-46**
210	W	SDRM	0.001	0.001	1.55	3.5E-02	8.5E-01	0.208	0.077	0.32	0.0E+00	**0.0E+00**
215	C	SDRM	0.001	0.001	0.92	3.9E-01	1.0E+00	0.009	0.004	0.42	8.3E-15	**1.2E-13**

215	D	SDRM	0.003	0.002	0.64	6.4E-04	**2.1E-02**	0.008	0.004	0.55	4.7E-08	**3.7E-07**
215	E	SDRM	0.001	0.002	1.25	1.1E-01	1.0E+00	0.002	0.001	0.75	1.2E-01	3.7E-01
215	F	SDRM	0.000	0.000	0.60	5.7E-01	1.0E+00	0.118	0.083	0.68	3.9E-42	**8.2E-41**
215	I	SDRM	0.000	0.000	1.05	5.1E-01	1.0E+00	0.022	0.020	0.94	1.4E-01	3.7E-01
215	S	SDRM	0.004	0.003	0.82	4.5E-02	1.0E+00	0.025	0.018	0.73	3.2E-08	**2.9E-07**
215	V	SDRM	0.000	0.000	0.80	5.8E-01	1.0E+00	0.009	0.005	0.57	6.8E-08	**4.8E-07**
215	Y	SDRM	0.000	0.000	0.09	2.6E-03	7.9E-02	0.305	0.134	0.35	0.0E+00	**0.0E+00**
219	E	SDRM	0.000	0.000	0.94	5.2E-01	1.0E+00	0.068	0.061	0.90	7.5E-04	**3.7E-03**
219	N	SDRM	0.000	0.000	0.34	1.1E-02	3.0E-01	0.034	0.014	0.42	4.5E-50	**1.0E-48**
219	Q	SDRM	0.001	0.001	0.72	7.8E-02	1.0E+00	0.126	0.069	0.51	5.9E-113	**1.6E-111**
219	R	SDRM	0.001	0.000	0.57	2.6E-02	6.9E-01	0.027	0.013	0.47	2.3E-33	**4.1E-32**
44	A	Candidate	0.000	0.000	3.59	2.5E-01	1.0E+00	0.017	0.006	0.33	1.8E-37	**3.1E-36**
65	N	Candidate	0.000	0.000	0.28	2.5E-02	4.2E-01	0.001	0.002	1.77	1.1E-02	5.2E-02
67	H	Candidate	0.000	0.000	3.59	2.5E-01	1.0E+00	0.002	0.001	0.55	2.8E-03	**2.5E-02**
67	S	Candidate	0.000	0.000	0.00	3.0E-01	1.0E+00	0.003	0.002	0.58	8.3E-04	**9.1E-03**
67	T	Candidate	0.000	0.000	0.00	1.0E+00	1.0E+00	0.002	0.002	0.63	1.0E-02	5.2E-02
69	A	Candidate	0.002	0.003	1.24	4.8E-02	7.1E-01	0.011	0.009	0.79	3.6E-03	**2.9E-02**
69	del	Candidate	0.000	0.000	0.00	5.4E-01	1.0E+00	0.001	0.003	2.30	9.4E-06	**1.1E-04**
69	G	Candidate	0.000	0.000	0.00	5.4E-01	1.0E+00	0.003	0.001	0.56	1.7E-03	**1.7E-02**
70	G	Candidate	0.000	0.000	0.00	1.0E+00	1.0E+00	0.005	0.003	0.72	8.6E-03	5.1E-02
70	N	Candidate	0.000	0.000	0.96	5.2E-01	1.0E+00	0.003	0.004	1.31	3.4E-02	1.0E-01
70	Q	Candidate	0.000	0.000	0.22	2.5E-02	4.2E-01	0.002	0.006	2.76	8.1E-13	**1.3E-11**
70	T	Candidate	0.000	0.000	1.05	5.1E-01	1.0E+00	0.003	0.007	2.25	1.6E-11	**2.2E-10**
215	A	Candidate	0.001	0.001	0.80	1.4E-01	1.0E+00	0.003	0.003	0.91	3.0E-01	4.6E-01
215	L	Candidate	0.001	0.001	1.05	4.8E-01	1.0E+00	0.002	0.002	0.84	2.3E-01	4.6E-01
215	N	Candidate	0.000	0.000	0.96	5.6E-01	1.0E+00	0.013	0.007	0.55	2.3E-12	**3.4E-11**
219	H	Candidate	0.000	0.000	0.00	1.0E+00	1.0E+00	0.003	0.001	0.41	8.4E-07	**1.1E-05**
219	W	Candidate	0.000	0.000	0.00	1.0E+00	1.0E+00	0.003	0.002	0.60	3.9E-03	**2.9E-02**

Estimated odds ratio (OR) by Fisher’s exact test, their p-values and adjusted p values by Holm’s method are shown. Significant increase or decrease in prevalence with adjusted p <0.05 is indicated in bold.

Table S7. NNRTI-associated SDRMs and candidate SDRMs: Comparisons of prevalence in RTI-naïve and NNRTI-experienced persons before and after 2009 using Fisher's exact test.

Pos	AA	SDRM	RTI-Naive					NNRTI-Experienced				
			% in <2009 (n=54,910)	% in ≥2009 (n=45,940)	OR	p	Adjusted p	% in <2009 (n=27,755)	% in ≥2009 (n=22,219)	OR	p	Adjusted p
100	I	SDRM	0.000	0.000	0.15	2.2E-03	3.5E-02	0.045	0.035	0.77	5.2E-09	**7.7E-08**
101	E	SDRM	0.001	0.002	1.35	3.2E-02	4.2E-01	0.072	0.093	1.34	5.3E-19	**9.0E-18**
101	P	SDRM	0.000	0.000	1.20	7.9E-01	1.0E+00	0.017	0.014	0.81	1.8E-03	**1.8E-02**
103	N	SDRM	0.013	0.016	1.24	2.2E-05	4.2E-04	0.358	0.356	0.99	3.4E-01	1.0E+00
103	S	SDRM	0.000	0.001	1.87	3.4E-02	4.2E-01	0.020	0.023	1.16	7.6E-03	6.1E-02
106	A	SDRM	0.000	0.000	0.51	1.2E-01	9.6E-01	0.018	0.014	0.76	7.1E-05	**9.2E-04**
106	M	SDRM	0.000	0.001	3.93	5.3E-04	9.5E-03	0.026	0.091	3.75	6.0E-224	**1.1E-222**
179	F	SDRM	0.000	0.000	0.30	9.4E-02	8.4E-01	0.003	0.002	0.76	8.7E-02	5.7E-01
181	C	SDRM	0.002	0.002	1.05	4.2E-01	1.0E+00	0.179	0.181	1.01	3.3E-01	1.0E+00
181	I	SDRM	0.000	0.000	2.39	2.6E-01	1.0E+00	0.008	0.006	0.73	2.4E-03	**2.2E-02**
181	V	SDRM	0.000	0.000	Inf	4.6E-01	1.0E+00	0.005	0.007	1.43	1.2E-03	**1.3E-02**
188	C	SDRM	0.000	0.000	0.80	3.9E-01	1.0E+00	0.007	0.010	1.44	2.0E-04	**2.4E-03**
188	H	SDRM	0.000	0.000	0.32	2.5E-02	3.5E-01	0.008	0.007	0.88	1.4E-01	6.9E-01
188	L	SDRM	0.000	0.001	1.56	5.5E-02	5.7E-01	0.045	0.044	0.98	3.7E-01	1.0E+00
190	A	SDRM	0.002	0.002	1.54	1.5E-03	2.6E-02	0.145	0.146	1.01	4.0E-01	1.0E+00
190	E	SDRM	0.000	0.001	2.72	3.3E-03	4.9E-02	0.005	0.006	1.20	8.2E-02	5.7E-01
190	S	SDRM	0.000	0.000	2.39	1.2E-01	9.6E-01	0.027	0.033	1.24	2.1E-05	**2.9E-04**
225	H	SDRM	0.000	0.000	0.36	5.1E-02	5.7E-01	0.043	0.074	1.79	6.1E-51	**1.1E-49**
230	L	SDRM	0.000	0.000	1.17	4.9E-01	1.0E+00	0.015	0.026	1.67	9.7E-16	**1.6E-14**
101	H	Candidate	0.000	0.000	Inf	4.3E-02	6.5E-01	0.013	0.013	1.00	5.2E-01	1.0E+00
101	N	Candidate	0.000	0.000	1.20	4.2E-01	1.0E+00	0.005	0.007	1.31	1.3E-02	1.0E-01
101	T	Candidate	0.000	0.000	1.59	2.7E-01	1.0E+00	0.004	0.002	0.64	5.3E-03	5.8E-02

138	K	Candidate	0.001	0.001	1.07	3.9E-01	1.0E+00	0.004	0.007	1.70	4.6E-06	**6.9E-05**
138	Q	Candidate	0.000	0.001	2.39	3.5E-03	6.3E-02	0.010	0.016	1.64	3.3E-10	**5.3E-09**
179	L	Candidate	0.000	0.000	0.80	4.9E-01	1.0E+00	0.002	0.003	1.90	4.2E-04	**5.0E-03**
179	M	Candidate	0.000	0.000	1.31	3.4E-01	1.0E+00	0.001	0.003	2.14	1.9E-04	**2.6E-03**
181	F	Candidate	0.000	0.000	0.85	5.1E-01	1.0E+00	0.002	0.002	0.90	3.3E-01	1.0E+00
188	F	Candidate	0.000	0.000	1.20	5.4E-01	1.0E+00	0.006	0.006	0.98	4.6E-01	1.0E+00
190	C	Candidate	0.000	0.000	0.00	1.0E+00	1.0E+00	0.001	0.001	1.11	3.9E-01	1.0E+00
190	Q	Candidate	0.000	0.000	0.00	1.0E+00	1.0E+00	0.003	0.004	1.10	2.8E-01	1.0E+00
221	Y	Candidate	0.001	0.001	0.95	4.5E-01	1.0E+00	0.065	0.081	1.27	5.8E-12	**9.9E-11**
227	C	Candidate	0.000	0.000	0.58	5.6E-01	1.0E+00	0.000	0.001	3.65	3.0E-04	**3.9E-03**
227	L	Candidate	0.001	0.000	0.55	3.4E-02	5.4E-01	0.022	0.035	1.62	1.1E-18	**1.9E-17**
234	I	Candidate	0.000	0.000	0.47	1.5E-01	1.0E+00	0.003	0.004	1.46	8.3E-03	7.5E-02
236	L	Candidate	0.000	0.000	1.30	2.9E-01	1.0E+00	0.001	0.002	1.95	6.0E-03	6.0E-02
238	N	Candidate	0.000	0.001	1.78	2.1E-02	3.6E-01	0.006	0.004	0.80	5.3E-02	3.2E-01
238	T	Candidate	0.001	0.000	0.79	2.5E-01	1.0E+00	0.027	0.030	1.11	3.1E-02	2.2E-01

Estimated odds ratio (OR) by Fisher's exact test, their p-values and adjusted p values by Holm's method are shown. Significant increase or decrease in prevalence with adjusted $p < 0.05$ is indicated in bold.

Table S8. PI-associated SDRMs and candidate SDRMs: Comparisons of prevalence in PI-naïve and PI-experienced persons before and after 2009 using Fisher's exact test.

Pos	AA	SDRM	PII-Naive					PI-Experienced				
			% in <2009 (n=61,067)	% in ≥2009 (n=54,813)	OR	p	Adjusted p	% in <2009 (n=22,266)	% in ≥2009 (n=10,534)	OR	p	Adjusted p
23	I	SDRM	0.000	0.000	1.00	5.7E-01	1.0E+00	0.013	0.011	0.80	2.1E-02	8.5E-02
24	I	SDRM	0.000	0.000	0.26	1.9E-02	6.9E-01	0.056	0.025	0.43	2.6E-40	**8.2E-39**
30	N	SDRM	0.000	0.000	1.25	4.1E-01	1.0E+00	0.062	0.024	0.38	6.4E-54	**2.0E-52**
32	I	SDRM	0.000	0.000	0.37	1.0E-01	1.0E+00	0.050	0.025	0.49	1.2E-27	**2.7E-26**
46	I	SDRM	0.003	0.003	1.31	8.0E-03	3.0E-01	0.212	0.126	0.54	2.9E-82	**9.7E-81**
46	L	SDRM	0.003	0.004	1.20	4.2E-02	1.0E+00	0.092	0.034	0.35	4.5E-88	**1.6E-86**
47	A	SDRM	0.000	0.000	0.00	1.0E+00	1.0E+00	0.004	0.010	2.43	7.8E-10	**1.2E-08**
47	V	SDRM	0.000	0.000	0.77	3.5E-01	1.0E+00	0.045	0.020	0.42	4.7E-34	**1.3E-32**
48	M	SDRM	0.000	0.000	0.00	1.0E+00	1.0E+00	0.005	0.002	0.28	2.2E-08	**3.0E-07**
48	V	SDRM	0.000	0.000	0.00	1.0E+00	1.0E+00	0.037	0.007	0.18	1.0E-67	**3.4E-66**
50	L	SDRM	0.000	0.000	0.00	4.1E-02	1.0E+00	0.015	0.025	1.70	1.9E-10	**3.0E-09**
50	V	SDRM	0.000	0.000	0.28	7.6E-02	1.0E+00	0.018	0.012	0.66	1.9E-05	**1.9E-04**
53	L	SDRM	0.000	0.000	0.72	2.3E-01	1.0E+00	0.059	0.030	0.49	7.3E-32	**1.8E-30**
53	Y	SDRM	0.000	0.000	2.23	3.4E-02	1.0E+00	0.004	0.002	0.35	9.9E-06	**1.1E-04**
54	A	SDRM	0.000	0.000	0.00	1.0E+00	1.0E+00	0.013	0.004	0.27	1.1E-18	**2.4E-17**
54	L	SDRM	0.000	0.000	0.56	2.5E-01	1.0E+00	0.030	0.014	0.45	3.4E-21	**7.6E-20**
54	M	SDRM	0.000	0.000	1.11	7.1E-01	1.0E+00	0.023	0.006	0.24	1.5E-35	**4.4E-34**
54	S	SDRM	0.000	0.000	0.00	1.0E+00	1.0E+00	0.007	0.001	0.21	1.1E-12	**1.9E-11**
54	T	SDRM	0.000	0.000	0.64	3.4E-01	1.0E+00	0.008	0.002	0.19	7.1E-16	**1.4E-14**
54	V	SDRM	0.000	0.000	1.67	3.1E-01	1.0E+00	0.231	0.120	0.46	1.2E-131	**4.4E-130**
73	A	SDRM	0.000	0.000	1.11	6.5E-01	1.0E+00	0.006	0.002	0.34	8.1E-08	**1.1E-06**
73	C	SDRM	0.000	0.000	1.67	4.5E-01	1.0E+00	0.011	0.003	0.30	7.5E-15	**1.4E-13**
73	S	SDRM	0.000	0.000	1.67	1.1E-01	1.0E+00	0.083	0.027	0.31	4.6E-92	**1.6E-90**

73	T	SDRM	0.000	0.000	0.00	1.0E+00	1.0E+00	0.024	0.006	0.25	6.0E-35	**1.7E-33**
76	V	SDRM	0.000	0.000	1.34	4.3E-01	1.0E+00	0.033	0.033	1.00	4.9E-01	4.9E-01
82	A	SDRM	0.000	0.000	0.96	5.1E-01	1.0E+00	0.220	0.122	0.49	2.7E-106	**1.0E-104**
82	C	SDRM	0.000	0.000	Inf	2.2E-01	1.0E+00	0.006	0.004	0.66	8.7E-03	**4.3E-02**
82	F	SDRM	0.000	0.000	12.25	1.8E-03	7.0E-02	0.018	0.011	0.59	1.9E-07	**2.3E-06**
82	L	SDRM	0.000	0.000	1.35	2.5E-01	1.0E+00	0.003	0.002	0.69	7.6E-02	2.3E-01
82	M	SDRM	0.000	0.000	Inf	2.2E-01	1.0E+00	0.003	0.004	1.28	1.2E-01	2.4E-01
82	S	SDRM	0.000	0.000	0.56	5.4E-01	1.0E+00	0.013	0.005	0.40	6.0E-12	**1.0E-10**
82	T	SDRM	0.000	0.000	0.00	1.0E+00	1.0E+00	0.028	0.008	0.27	8.2E-38	**2.5E-36**
83	D	SDRM	0.000	0.000	1.62	1.5E-01	1.0E+00	0.009	0.006	0.69	4.1E-03	**2.5E-02**
84	A	SDRM	0.000	0.000	0.00	1.0E+00	1.0E+00	0.001	0.000	0.08	2.8E-04	**1.9E-03**
84	C	SDRM	0.000	0.000	0.00	1.0E+00	1.0E+00	0.001	0.000	0.00	6.2E-05	**5.0E-04**
84	V	SDRM	0.000	0.000	1.98	7.0E-02	1.0E+00	0.122	0.038	0.28	6.8E-151	**2.7E-149**
85	V	SDRM	0.002	0.002	0.95	4.1E-01	1.0E+00	0.046	0.021	0.44	6.9E-33	**1.8E-31**
88	D	SDRM	0.000	0.000	0.67	2.3E-01	1.0E+00	0.054	0.027	0.50	3.4E-29	**8.0E-28**
88	S	SDRM	0.000	0.000	0.00	1.3E-04	5.1E-03	0.016	0.010	0.64	2.1E-05	**1.9E-04**
90	M	SDRM	0.002	0.002	0.97	4.2E-01	1.0E+00	0.296	0.096	0.25	0.0E+00	**0.0E+00**
10	F	Candidate	0.002	0.002	1.01	4.9E-01	1.0E+00	0.086	0.067	0.76	1.5E-09	**2.0E-08**
10	R	Candidate	0.000	0.000	0.74	3.8E-01	1.0E+00	0.004	0.002	0.59	1.1E-02	**4.2E-02**
11	L	Candidate	0.000	0.000	0.67	1.4E-01	1.0E+00	0.007	0.002	0.36	2.7E-07	**2.7E-06**
20	T	Candidate	0.001	0.001	0.91	3.4E-01	1.0E+00	0.053	0.041	0.77	1.8E-06	**1.6E-05**
24	F	Candidate	0.000	0.000	0.15	2.1E-05	3.6E-04	0.006	0.003	0.47	5.0E-05	**3.0E-04**
34	Q	Candidate	0.001	0.001	1.01	5.2E-01	1.0E+00	0.026	0.013	0.49	1.8E-15	**2.7E-14**
41	T	Candidate	0.000	0.000	1.11	4.0E-01	1.0E+00	0.001	0.001	1.27	3.2E-01	5.4E-01
43	T	Candidate	0.001	0.001	0.92	3.4E-01	1.0E+00	0.056	0.030	0.52	3.9E-27	**6.6E-26**
46	V	Candidate	0.000	0.001	1.44	9.4E-02	1.0E+00	0.005	0.004	0.84	1.8E-01	5.4E-01
48	A	Candidate	0.000	0.000	2.23	4.6E-01	1.0E+00	0.005	0.002	0.36	9.4E-06	**7.5E-05**
66	V	Candidate	0.001	0.001	0.87	2.8E-01	1.0E+00	0.011	0.004	0.40	4.5E-10	**6.3E-09**
71	I	Candidate	0.001	0.001	1.34	7.6E-02	1.0E+00	0.032	0.017	0.51	4.4E-17	**7.0E-16**
71	L	Candidate	0.000	0.000	0.00	2.8E-01	1.0E+00	0.004	0.002	0.58	7.7E-03	**3.8E-02**

74	P	Candidate	0.001	0.000	0.07	9.3E-08	1.7E-06	0.019	0.013	0.67	1.5E-05	**1.1E-04**
88	G	Candidate	0.000	0.000	0.00	1.0E+00	1.0E+00	0.002	0.001	0.78	2.6E-01	5.4E-01
89	T	Candidate	0.000	0.000	1.86	3.1E-01	1.0E+00	0.004	0.009	2.21	3.0E-08	**3.3E-07**
89	V	Candidate	0.001	0.000	0.70	8.9E-02	1.0E+00	0.037	0.014	0.37	1.9E-34	**3.4E-33**
93	M	Candidate	0.001	0.001	1.10	3.1E-01	1.0E+00	0.007	0.002	0.33	1.0E-08	**1.2E-07**

Estimated odds ratio (OR) by Fisher's exact test, their p-values and adjusted p values by Holm's method are shown. Significant increase or decrease in prevalence with adjusted p <0.05 is indicated in bold.

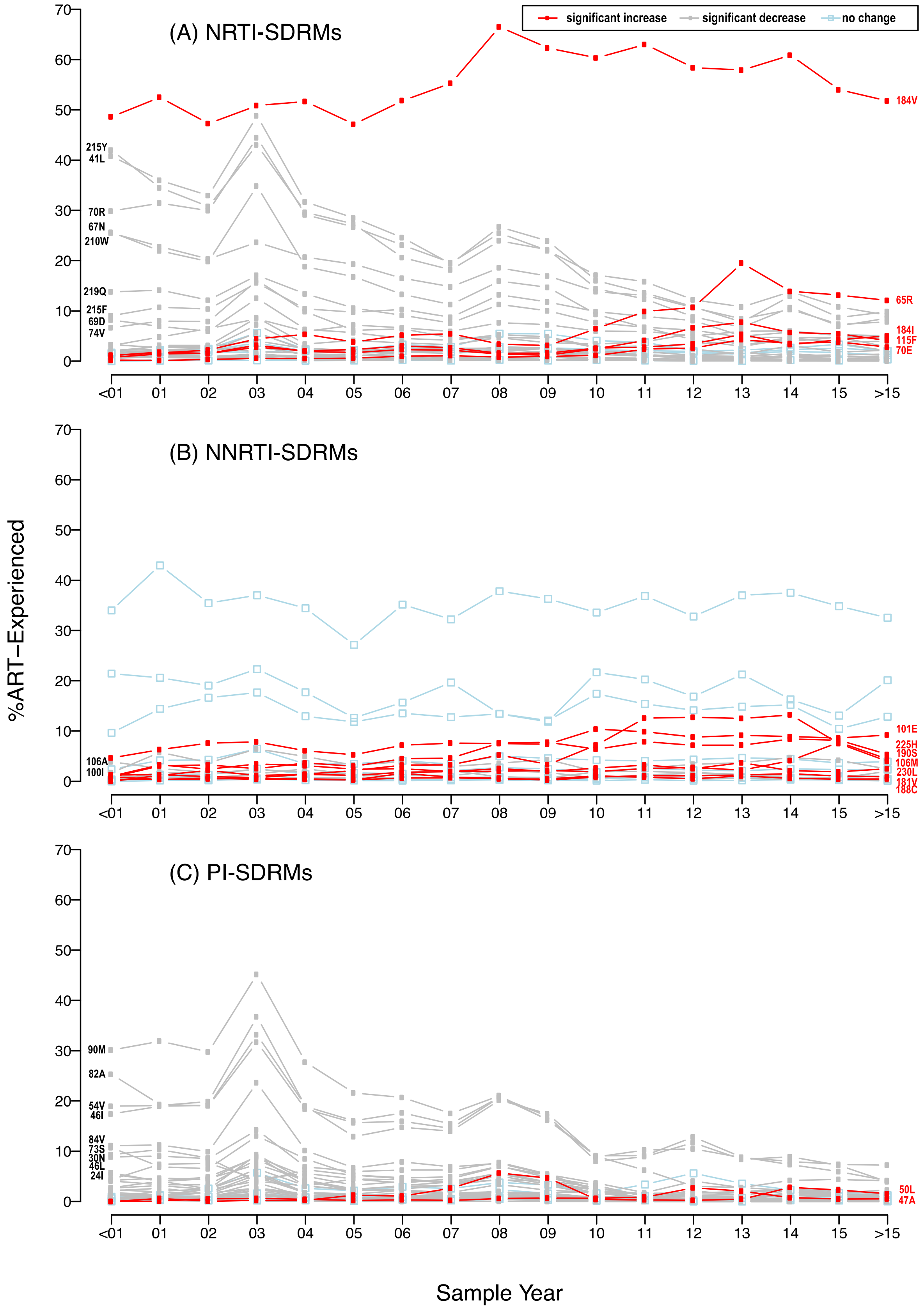
significant increase
significant decrease
no change
(A) NRTI-SDRMs
215Y
41L
70R
67N
210W
219Q
215F
69D
74V
184V
65R
184I
115F
70E
(B) NNRTI-SDRMs
106A
100I
101E
225H
190S
106M
230L
181V
188C
(C) PI-SDRMs
90M
82A
54V
46I
84V
73S
30N
46L
24I
50L
47A
%ART−Experienced
Sample Year
<01
01
02
03
04
05
06
07
08
09
10
11
12
13
14
15
>15

Figure S1. Yearly prevalence of (A) NRTI-SDRMs in NRTI-experienced persons, (B) NNRTI-SDRMs in NNRTI-experienced persons and (C) PI-SDRMs in PI-experienced persons. SDRMs significantly increased in prevalence are shown in red and indicated in red font on the right of the plotted lines. SDRMs significantly decreased in prevalence are shown in grey and indicated in black font on the left of the plotted lines. SDRMs did not change significantly in prevalence are shown in light blue. For SDRMs that decreased with a very low overall prevalence or did not change in prevalence, the labels are not shown. Full lists of NRTI-SDRMs, NNRTI-SDRMs and PI-SDRMs according to the change in prevalence are shown in Table 1, 3, and 5, respectively.

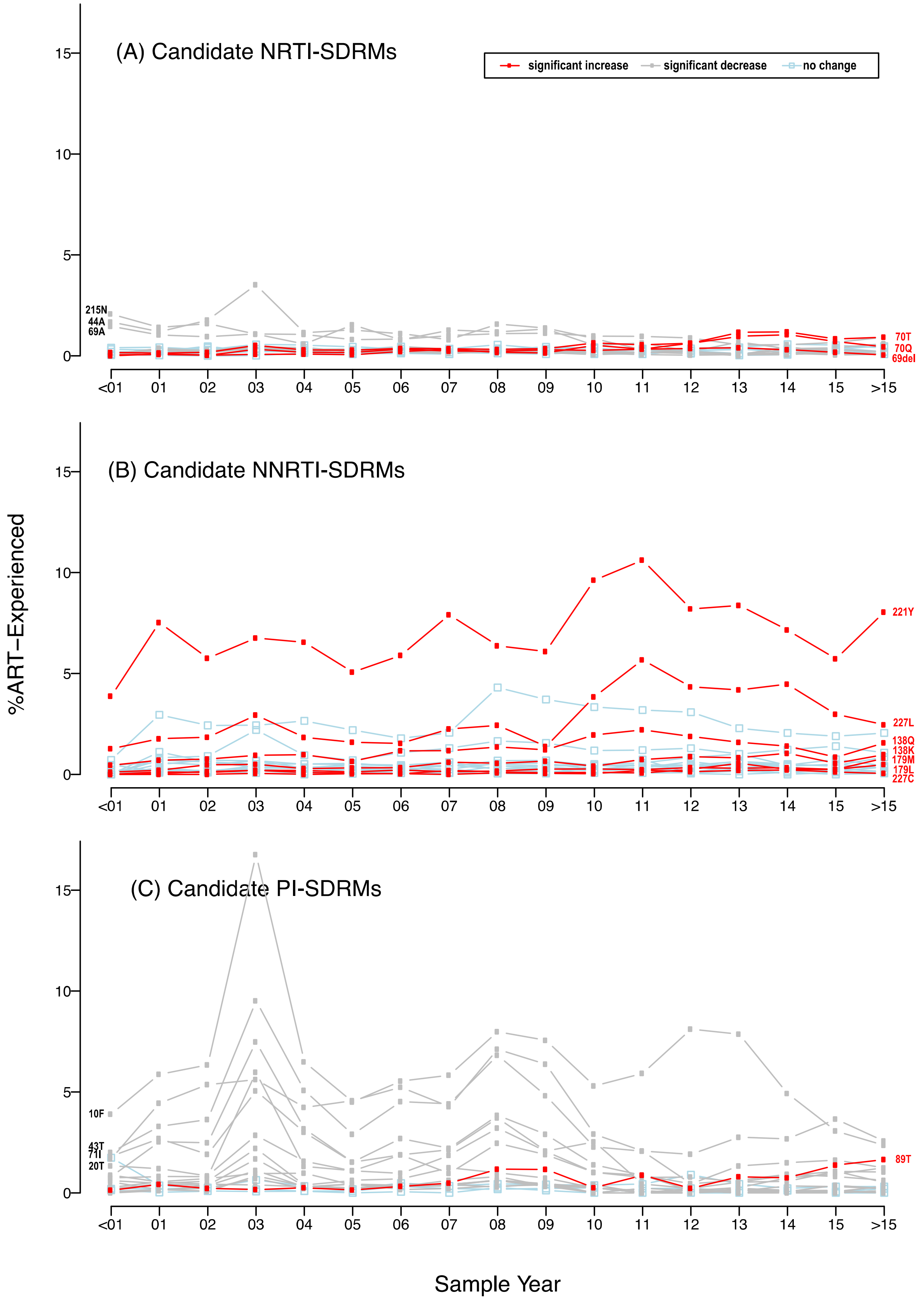

(A) Candidate NRTI-SDRMs
significant increase
significant decrease
no change
215N
44A
69A
70T
70Q
69del
(B) Candidate NNRTI-SDRMs
221Y
227L
138Q
138K
179M
179L
227C
(C) Candidate PI-SDRMs
10F
43T
71I
20T
89T
%ART−Experienced
Sample Year
0
5
10
15
<01
01
02
03
04
05
06
07
08
09
10
11
12
13
14
15
>15

Figure S2. Yearly prevalence of (A) candidate NRTI-SDRMs in NRTI-experienced persons, (B) candidate NNRTI-SDRMs in NNRTI-experienced persons and (C) candidate PI-SDRMs in PI-experienced persons. Candidate SDRMs significantly increased in prevalence are shown in red and indicated in red font on the right of the plotted lines. Candidate SDRMs significantly decreased in prevalence are shown in grey and indicated in black font on the left of the plotted lines. Candidate SDRMs did not change significantly in prevalence are shown in light blue. For candidate SDRMs that decreased with a very low overall prevalence or did not change in prevalence, the labels are not shown. Full lists of candidate NRTI-SDRMs, NNRTI-SDRMs and PI-SDRMs according to the change in prevalence are shown in Table 2, 4, and 6, respectively.
